# Novel hemostatic adhesive powder to prevent delayed bleeding after endoscopic submucosal dissection in the GI tract: first U.S. multicenter experience

**DOI:** 10.1016/j.igie.2024.10.002

**Published:** 2024-10-18

**Authors:** Dennis Yang, Amit Bhatt, Maham Hayat, Muhammad K. Hasan, Hiroyuki Aihara

**Affiliations:** 1 Center for Interventional Endoscopy, AdventHealth, Orlando, Florida, USA; 2 Department of Gastroenterology and Hepatology, Cleveland Clinic, Cleveland, Ohio, USA; 3 Division of Gastroenterology, Hepatology and Endoscopy, Brigham and Women’s Hospital, Harvard Medical School, Boston, Massachusetts, USA

## Abstract

**Background and Aims:**

Delayed bleeding (DB) is a common adverse event after endoscopic submucosal dissection (ESD). We evaluated a novel hemostatic powder (UI-EWD, Nexpowder; Medtronic, Minneapolis, Minn, USA) to prevent DB after ESD.

**Methods:**

This was a multicenter retrospective analysis of ESDs performed between January 2023 and March 2024 in which UI-EWD was applied to prevent DB. Cases in which endoscopic closure of the post-ESD mucosal defect was performed were excluded. DB was defined as a bleeding event requiring hospitalization, blood transfusion, or any intervention within 30 days after the procedure. Technical success of UI-EWD was defined as successful delivery and application of the hemostatic powder over the entire mucosal defect.

**Results:**

Eighty-three patients (median age, 66 years) underwent ESD in the esophagus (n = 18), stomach (n = 15), colon (n = 38), and rectum (n = 12). The median lesion size was 50 mm (interquartile range, 41-70 mm). UI-EWD was successfully applied in all defects, although in 2 cases (2.4%) a second delivery catheter had to be used to complete the procedure. DB occurred in 3 patients (3.6%): 2 after gastric ESD and 1 after colonic ESD within 24 hours of the index procedure. None required intervention on repeat endoscopy. There were no cases of perforation. En bloc and R0 resection rates were 96.2% and 88.7%, respectively.

**Conclusions:**

UI-EWD can be easily applied to mucosal defects after ESD throughout the GI tract. Initial data from this multicenter study demonstrate that the use of UI-EWD was associated with a relatively low rate of DB after ESD. Additional comparative studies are needed to corroborate these preliminary findings.

Endoscopic submucosal dissection (ESD) has become a widely accepted modality for selected superficial GI neoplasms in both the upper and lower GI tract.^1^^,^^2^ Nonetheless, ESD can be associated with serious adverse events (AEs), including delayed bleeding (DB), which has been reported to range from 1.8% to as high as 15.6% depending on several patient- and lesion-related factors.^3^ Prophylactic closure of the post-ESD mucosal defect has been shown to potentially reduce the risk of DB; however, this approach is not always technically feasible depending on the location and size of the defect.^3^^,^^4^

The introduction of hemostatic topical agents over recent years has expanded our endoscopic armamentarium for hemostasis.^5^ Theoretically, hemostatic agents have the advantage of ease of use and ability to cover a large surface area that may be challenging to treat with other strategies, including mechanical closure. A novel hemostatic adhesive powder (UI-EWD, Nexpowder; Medtronic, Minneapolis, Minn, USA) was recently approved by the U.S. Food and Drug Administration for endoscopic hemostasis. UI-EWD consists of a biocompatible natural polymer produced using aldehyde dextran and succinic acid modified e-poly (l-lysine) that immediately transforms into a highly adhesive hydrogel on contact with fluid. Currently, no data are available on the role of UI-EWD for bleeding prophylaxis. The aim of this multicenter retrospective study was to evaluate the feasibility of applying UI-EWD on mucosal defects after ESD in the GI tract and its efficacy in the prevention of DB.

## Methods

### Study design and population

This was a retrospective multicenter cohort study of consecutive patients aged ≥18 years who underwent UI-EWD application after ESD for GI lesions at 3 centers in the United States between January 2023 and March 2024. Cases were excluded if mucosal defect closure was partially or completely achieved, defined as some or complete tissue apposition of the resection margins after ESD.

All patients provided informed consent for the procedures. The study was approved by the institutional review board at each participating institution, with the Center for Interventional Endoscopy at AdventHealth Orlando serving as the clinical coordinating center. All authors had access to the study data and reviewed and approved the final manuscript.

### Procedures

#### Endoscopic submucosal dissection

All ESD procedures were performed by experienced interventional endoscopists from each participating institution. All procedures were performed with patients under intravenous moderate sedation, deep sedation, or general anesthesia with endotracheal intubation, at the discretion of the participating center. Carbon dioxide was used for insufflation in all cases. Target lesions were examined under high-definition white-light endoscopy and digital or dye-based chromoendoscopy and categorized according to their morphology.^6^

ESD was performed as previously described.^2^ Briefly, the submucosa was expanded by injection of a mixture of a blue-colored dye and viscous solution to create a submucosal lift. After this, ESD was carried out using a needle-type knife (Hybrid knife [Erbe USA, Marietta, Ga, USA], Proknife [Boston Scientific, Marlborough, Mass, USA], Dual J knife [Olympus America, Center Valley, Pa, USA]) and/or insulated-tip knife (IT-2, IT-nano; Olympus America).

#### UI-EWD application after ESD

**Figure 1:**
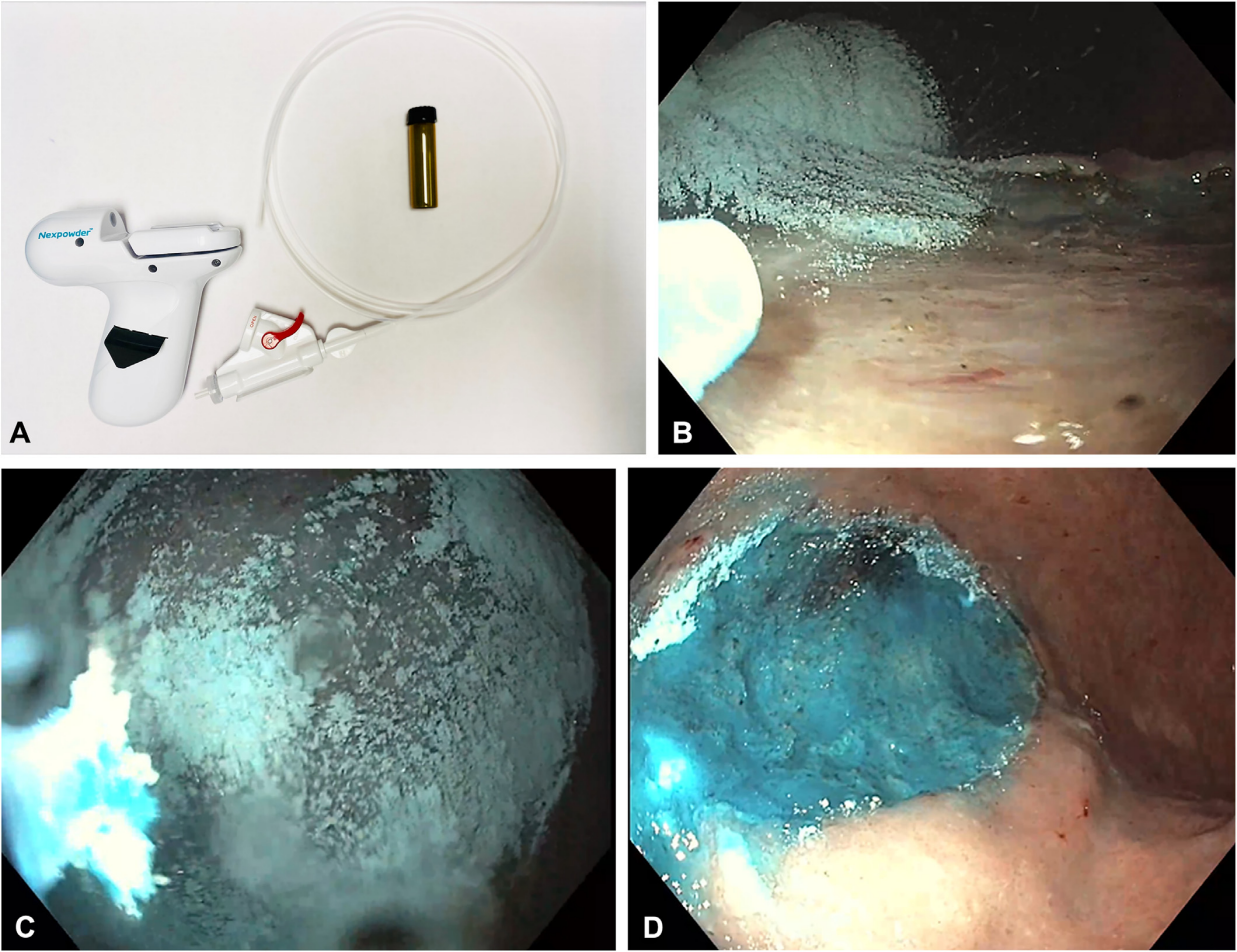
**A,** UI-EWD hemostatic system with powder vial, spray body, and delivery catheter. **B** and **C,** After endoscopic submucosal dissection (ESD), the UI-EWD hemostatic powder is sprayed over the entire ESD mucosal defect under direct visualization. **D,** The hemostatic powder immediately forms an adhesive hydrogel over the tissue on contact with fluid.

After ESD, UI-EWD was applied to the entire mucosal defect under direct visualization using the delivery system, which consisted of the powder vial connected to a spray body and catheter (Fig. 1A). For UI-EWD application, the catheter attached to the spray body was inserted through the accessory channel of the endoscope or colonoscope. The tip of the catheter was placed 1 to 2 cm from the target, and the powder was delivered with the goal of coating the entire mucosal defect, resulting in the formation of an adhesive gel (Fig. 1B-D, Video 1, available online at www.igiejournal.org). UI-EWD was applied with a maximum release of 6 g of powder. All mucosal defects were left open without endoscopic closure. Coagulation of visible nonbleeding vessels at the base of the ESD defect was performed at the discretion of the endoscopist on a case-by-case basis. All patients who underwent esophageal or gastric ESD were maintained on high-dose proton pump inhibitors for at least 2 to 4 weeks.

### Outcome measures and definitions

The primary outcome of this study was the rate of DB. DB was defined as a bleeding event that required a visit to the emergency department, hospitalization, blood transfusion, endoscopy, surgery, or any other invasive intervention to control bleeding and that occurred postprocedurally and within 30 days after the procedure. Antithrombotic use was defined as use of an antiplatelet agent within 7 days of the resection or an anticoagulant (warfarin or novel anticoagulant) within 5 days of the resection and/or reinitiating these medications within 7 days after the procedure. The secondary outcome was the technical success of UI-EWD, defined as successful delivery and application of UI-EWD over the entire mucosal defect confirmed by endoscopic visualization.

AEs were graded according to a standardized AE classification system for GI endoscopy (AGREE).^7^ ESD time was defined as the time interval from initiation of submucosal injection or margin marking (whichever occurred first) to completion of the ESD procedure followed by UI-EWD application. Total procedure time was defined as the time elapsed between scope insertion to withdrawal from the patient. En bloc resection was defined as excision of the visible targeted lesion in a single specimen. Complete resection (R0) was defined as en bloc resection with lateral and deep margins free of neoplasia on histologic evaluation.

### Statistical analysis

Descriptive statistics for each baseline variable was obtained and are expressed as mean and standard deviation or median and interquartile range (IQR). All statistical analysis was performed with the open source statistical software package R (version 3.5.0).

## Results

### Baseline and procedural characteristics

Ninety-one patients underwent ESD followed by UI-EWD application of the mucosal defect, of which 8 patients were excluded because either a partial (n = 6) or complete (n = 2) mechanical closure of the mucosal defect was performed. Patient and procedural characteristics are shown in Table 1.

**Table 1:** Patient and procedural characteristics

Characteristics	Values
Patient	
Age, y	66 (56-70)
Female sex	43 (52.4)
Race or ethnicity	
White	64 (77.1)
African American	5 (6.0)
Hispanic	8 (9.6)
Asian	6 (7.2)
American Society of Anesthesiologists grade	
I	25 (30.1)
II	34 (41)
III	24 (28.9)
IV	0
Antithrombotic use	
Aspirin	35 (42.7)
Clopidrogrel	8 (9.6)
Warfarin	10 (12.0)
Apixaban	16 (19.3)
Prior incomplete EMR attempt	20 (24.1)
Lesion location	
Esophagus	18 (21.7)
Stomach	15 (18.1)
Colon	38 (45.8)
Rectum	12 (14.5)
Procedural	
Lesion size, mm	50 (41-70)
Paris classification	
Is	8 (9.6)
IIa	45 (54.2)
IIa+Is	13 (15.7)
IIa+IIc	14 (16.9)
Endoscopic submucosal dissection time, min	65 (60-75)
Total procedure time, min	86 (80-97)
Resection outcomes	
En bloc resection	75 (90.4)
R0 (complete) resection	70 (84.3)
Histopathology	
Esophagus	
Barrett’s adenocarcinoma	9 (10.8)
Barrett’s high-grade dysplasia	7 (8.4
Granular cell tumor	1 (1.2)
Leiomyoma	1 (1.2)
Stomach	
Adenocarcinoma	5 (6.0)
High-grade dysplasia	5 (6.0)
Neuroendocrine tumor	4 (4.8)
Hyperplastic	1 (1.2)
Colon	
Adenoma with low-grade dysplasia	12 (14.5)
Adenoma with high-grade dysplasia	16 (19.3)
Adenocarcinoma	10 (12.0)
Rectum	
Adenoma with low-grade dysplasia	3 (3.6)
Adenoma with high-grade dysplasia	5 (6.0)
Adenocarcinoma	4 (4.8)

Values are median (interquartile range) or n (%).

Eighty-three patients (median age, 66 years; 52.4% women) were included for analysis and underwent ESD in the esophagus (n = 18), stomach (n = 15), colon (n = 38), and rectum (n = 12). The proportion of patients on antiplatelet and anticoagulant medications was 49.4% (n = 41) and 31.3% (n = 26), respectively. All patients resumed their antithrombotic agents within 24 to 48 hours after the ESD. The median lesion size was 50 mm (IQR, 41-70), and the median ESD and procedure times were 65 minutes (IQR, 60-75) and 86 minutes (IQR, 80-97), respectively. En bloc and R0 resection were achieved in 96.2% and 88.7%, respectively. The final ESD histopathology is summarized in Table 1.

### UI-EWD application and AEs

UI-EWD was successfully applied in all cases. However, in 2 cases (2.4%), after colonic ESDs in the ascending colon, the catheters were kinked during insertion, which resulted in clogging during powder delivery. Final UI-EWD delivery and application were successfully achieved in both cases by straightening the colonoscope, reinserting a second catheter, and then advancing to the site of the mucosal defect for UI-EWD application.

In aggregate, DB occurred in 3 patients (3.6%) (Table 2). In all 3 patients, bleeding occurred within 24 hours after ESD in the antrum of the stomach (n = 2) and cecum (n = 1). In all 3 cases bleeding had ceased spontaneously by the time of repeat endoscopy within 24 to 48 hours from the time of presentation. All patients recovered without any sequelae. No cases of perforation were reported.

**Table 2:** Patients with delayed bleeding after endoscopic submucosal dissection and UI-EWD application

Patient no.	Age (y)	Sex	American Society of Anesthesiologists class	Antithrombotics	Lesion location	Lesion size (mm)	Paris classification	Endoscopic submucosal dissection procedure time (min)	Histopathology	Onset of delayed bleeding	Outcome
1	36	M	I	No	Stomach	120	IIa	115	Well-differentiated adenocarcinoma	Within 24 h	Ceased spontaneously
2	64	F	III	Yes	Stomach	100	IIa	127	High-grade dysplasia	Within 24 h	Ceased spontaneously
3	75	M	II	Yes	Cecum	65	Is	94	High-grade dysplasia	Within 24 h	Ceased spontaneously

*UI-EWD*, Novel hemostatic powder.

## Discussion

DB is the most common AE after ESD. This multicenter study reported an initial experience on the use of the UI-EWD hemostatic powder for DB prophylaxis after ESD in the upper and lower GI tract. Our preliminary results show that UI-EWD was easy to use and associated with high technical success and an overall low rate of DB.

Prophylactic closure of mucosal defects after endoscopic resection has been shown to reduce the risk of bleeding.^4^ Large lesion size is a recognized risk factor for DB after ESD, yet complete closure of mucosal defects with clips alone, particularly for lesions ≥40 mm in size, is technically challenging and can only be accomplished in 57% to 68% of cases.^8^^,^^9^ Notably, partial instead of complete closure of defects does not appear to be protective against DB, and thereby its clinical utility is questionable.^9^ Although novel tissue apposition devices have increased our ability to close larger mucosal defects, data on their optimal use and cost-effectiveness remain limited.^10^^,^^11^

Hemostatic topical agents have garnered attention as alternatives to address the limitations in conventional endoscopic techniques for the management of GI bleeding. Several agents, such as TC-325 (Hemospray; Cook Medical, Bloomington, Ind, USA) and an absorbable modified polymer derived from plant starch (EndoClot; Olympus America), have been studied in the setting of primary, rescue, and combination treatments with conventional methods for the treatment of GI bleeding.^5^ On contact with blood, these agents rapidly absorb water and concentrate coagulation factors.

As opposed to these agents, UI-EWD does not require clot formation to induce hemostasis, and active bleeding is not necessary for UI-EWD to be effective, theoretically making it ideal as a topical agent for bleeding prophylaxis. UI-EWD consists of a polymer that converts to a highly adhesive hydrogel on contact with fluid, thereby providing a mechanical barrier over an ulcer bed. In a study evaluating the performance of UI-EWD for the management of upper GI nonvariceal bleeding, Park et al^12^ noted that the 30-day recurrent bleeding rate among patients who achieved hemostasis was only 3.7% (2/54). It has been speculated that the reaction between the hydrogel and tissue translates into an extended barrier that potentially provides more-effective tissue sealing and mechanical tamponade.^13^

In this study, the overall DB rate after ESD was 3.6%. The relatively low incidence of DB is particularly remarkable if we consider the median size of the lesion was 50 mm and that only cases in which endoscopic closure could not be achieved or performed were included in the analysis. Notably, the 3 cases of DB occurred after ESD of lesions >6 cm, with large size being a known factor associated with higher risk of bleeding.^14^ In all 3 cases, repeat endoscopy was performed within 24 hours from the index procedure. On examination, clean base ulcers were identified without active bleeding, but the hydrogel barrier was no longer appreciated over the defect. A study had identified that the UI-EWD remains present in 70% of cases by second-look endoscopy at 24 hours.^15^ We presume that different factors (ie, location within the GI tract, peristalsis, size of the mucosal defect, oral intake) may impact the adherence and durability of the gel. Additional data are needed to identify factors that influence the stability of the hydrogel and thereby its potential effectiveness in hemostasis.

In aggregate, our results show that UI-EWD was safe and easy to use. Application of older iterations of hemostatic powders was limited by several technical issues, including frequent clogging of the delivery catheter and impaired visualization after powder administration secondary to the formation of powder-in-air suspensions.^14^^,^^15^ Successful delivery of the UI-EWD was achieved in all cases in this study without impaired visualization. In 2 cases, because of looping of the colonoscope, kinking of the delivery catheter occurred during insertion. In our experience, straightening the scope and inserting the delivery catheter through the open biopsy valve facilitates advancement of the catheter and delivery of the UI-EWD.

We acknowledge the limitations of this study. The study was retrospective and limited by its uncontrolled design. Because this study only reported consecutive cases in which UI-EWD was used after ESD, we did not capture data on the number of cases, procedural characteristics, or reasons for which UI-EWD may have not been used during the study period, rendering selection bias. DB events and other AEs were identified by reviewing the electronic chart and available medical records. Hence, there is a risk that procedure-related AEs were under-captured and/or under-reported. Nonetheless, this potential drawback is somewhat mitigated by the availability of Epic Systems Corporation, Wisconsin, USA electronic health record system at the participating institutions, which facilitates automatic distribution and exchange of patient information even through different electronic health record systems. Furthermore, all post-ESD patients were routinely contacted by the respective endoscopic units within 24 to 48 hours of their procedure to assess for delayed AEs, which corresponds to the time window with the highest risk for DB. We acknowledge that cases were performed at centers specializing in ESD; thus, results may not be generalizable. Finally, this was a single-arm retrospective observational study. In the absence of a control group, it is difficult to determine the impact of our intervention, and it remains uncertain whether UI-EWD truly reduced the risk of DB after ESD. Nonetheless, our initial findings have important clinical implications in that they support the safety and feasibility of applying UI-EWD in post-ESD mucosal defects throughout the GI tract and provide a reference framework for the rate of DB with UI-EWD for sample size calculations ideally in future randomized trials.

In conclusion, this initial multicenter experience demonstrates that UI-EWD can be easily applied to mucosal defects after ESD through the GI tract. The use of UI-EWD was associated with a relatively low rate of DB. Future studies are needed to determine whether the risk of DB is reduced in all patients receiving UI-EWD prophylactically and how it may compare with other established modalities to prevent DB both from a cost and performance effectiveness standpoint.

## Disclosure

The following authors disclosed financial relationships: D. Yang: Consultant for Olympus, Fujifilm, Boston Scientific, Medtronic, 3D-Matrix, Microtech, and Neptune Medical; research support from Microtech and 3D-Matrix. A. Bhatt: Consultant for Boston Scientific, Steris, and Medtronic; royalties from Medtronic. M. K. Hasan: Consultant for Boston Scientific, Olympus, and Microtech. H. Aihara: Consultant for Boston Scientific, Olympus America, Fujifilm, Medtronic, Auris Health, Lumendi, ConMed, and 3-D Matrix; advisory board Endoquest Robotics; research support from Boston Scientific. All other authors disclosed no financial relationships.

## Supplementary data

Video 1 Mucosal defect on the greater curvature of the stomach after gastric endoscopic submucosal dissection. The UI-EWD hemostatic powder is sprayed over the entire defect with formation of an adhesive hydrogel.
